# Next-Generation Antimicrobial Peptides for Biofilm-Associated Infections: Engineering, Biomaterial Delivery and AI-Assisted Discovery

**DOI:** 10.3390/antibiotics15090880

**Published:** 2026-09-08

**Authors:** Aghilas Akkache, Mattéo Védère, Skander Hathroubi

**Affiliations:** SPARTHA Medical, Centre de Recherche en Biomédecine de Strasbourg, 1 Rue Eugène Boeckel, 67000 Strasbourg, France; aakkache@sparthamedical.eu (A.A.); vedere.matteo.pro@gmail.com (M.V.)

**Keywords:** antimicrobial peptides, biofilms, hydrogel coatings, peptide engineering, AI, machine learning, generative models, peptide antibiotics, biomaterials

## Abstract

Antimicrobial peptides (AMPs) are increasingly regarded as next-generation antimicrobial agents because of their broad-spectrum activity, rapid killing, antibiofilm potential, immunomodulatory properties, and mechanisms of action that differ from those of many conventional antibiotics. Despite these advantages, their clinical translation remains limited by proteolytic instability, hemolysis or cytotoxicity, poor pharmacokinetics, salt and serum sensitivity, production costs, and delivery challenges. The AMP field is therefore shifting from natural peptide discovery toward integrated engineering pipelines that combine rational peptide modification, biomaterial-based delivery, high-throughput screening, and artificial intelligence (AI), particularly machine learning (ML) and deep learning approaches. Chemical and structural modifications, including D-amino acid substitution, N-glycine substitution, cyclization, lipidation, PEGylation, terminal amidation, hydrocarbon stapling, hybridization, sequence truncation, metal coordination, and biomaterial immobilization, are being used to improve stability, potency, selectivity, antibiofilm activity, and tissue localization. In parallel, AI-guided approaches, including ML, deep learning, and generative modeling, enable large-scale exploration of diverse peptide sources, including microbiomes and extinct proteomes, to entirely new sequences, while supporting optimization of potency, selectivity, stability, toxicity, and synthesizability. This focused review summarizes recent advances in AMP engineering, biomaterial-assisted delivery, and AI-guided discovery for biofilm-associated infections in the context of antimicrobial resistance.

## 1. Introduction

The global rise of antimicrobial resistance has intensified the search for therapeutic molecules that act beyond the mechanisms of conventional antibiotics. The World Health Organization identifies antimicrobial resistance (AMR) as one of the top global public health and development threats. Recent global burden analyses estimated that bacterial AMR was directly responsible for approximately 1.14 million deaths worldwide in 2021 and was associated with 4.71 million deaths [1]. Recent WHO surveillance further confirms the scale of the problem: the WHO Global Antibiotic Resistance Surveillance Report 2025 analyzed more than 23 million bacteriologically confirmed infections reported by over 100 countries and highlighted persistent resistance among major pathogens causing bloodstream, urinary tract, gastrointestinal, and gonococcal infections [2]. Together, these data highlight the need for alternative antimicrobial molecules that can complement or bypass conventional antibiotic mechanisms.

Antimicrobial peptides (AMPs), also referred to as host-defense peptides in many biological contexts, represent one of the oldest and most conserved antimicrobial strategies in nature. Most AMPs are short peptides, commonly 10 to 50 amino acids in length, although AMP databases and reviews include broader ranges depending on peptide origin, structure, and definition. They are often enriched in positively charged and hydrophobic residues, giving many AMPs an amphipathic character that favors electrostatic interaction with negatively charged bacterial membranes. Historically, peptide antimicrobials entered modern microbiology through the discovery of gramicidins from soil *Bacillus*/*Brevibacillus* species in 1939, followed by gramicidin S in the 1940s, one of the first cyclic peptide antibiotics used clinically for wound infections [3,4]. Later discoveries, including defensins, cecropins, magainins, and cathelicidins, expanded the concept of AMPs from microbial antibiotics to ubiquitous components of innate immunity across plants, insects, amphibians, mammals, and humans [5,6,7,8]. Despite these early discoveries, the therapeutic development of AMPs remained relatively limited for several decades, as conventional small-molecule antibiotics became the dominant anti-infective strategy, and many peptide candidates were constrained by proteolytic instability, toxicity, poor pharmacokinetics, and manufacturing costs. Interest in AMPs has been revived in the context of increasing antimicrobial resistance, persistent biofilm-associated infections, and the need for antimicrobial strategies with mechanisms distinct from classical antibiotics. This renewed attention is also supported by the multifunctional nature of AMPs, which may combine direct antimicrobial activity with additional functions such as immunomodulation, endotoxin neutralization, wound-healing support, antibiofilm activity, and synergy with existing antibiotics [9,10].

Recent primary studies support this renewed interest. Zhang et al. reported an amphipathic peptide with activity against multidrug-resistant *Staphyloccoccus aureus* and associated biofilms, while Alexander et al. identified microbiome-derived AMPs with therapeutic activity against the WHO-priority pathogen *Acinetobacter baumannii* [11,12]. Unlike many small-molecule antibiotics that act through highly specific enzymatic or biosynthetic targets, AMPs frequently act through physicochemical interactions with microbial membranes, making resistance development more complex, although not impossible [13,14]. Recent reviews continue to emphasize this duality: AMPs may display a lower propensity for classical single-target resistance than conventional antibiotics, but bacteria can nevertheless evolve tolerance through envelope remodeling, surface charge modification, protease production, efflux, stress-response regulation, and biofilm-associated protection [15,16,17].

Once bound to the microbial surface, AMPs may induce membrane permeabilization, pore formation, membrane thinning, lipid clustering, depolarization, or detergent-like disruption [5,18,19]. Recent structural and mechanistic studies have refined this view by showing that membrane disruption is often dynamic and context-dependent, influenced by peptide concentration, lipid composition, peptide aggregation, secondary structure, and bacterial envelope architecture [16,20].

However, AMPs should not be understood only as membrane-disrupting agents. Many peptides also cross the membrane and interfere with intracellular processes, including DNA, RNA, protein synthesis, cell-wall synthesis, metabolic pathways, and stress-response systems. Indeed, in addition to causing membrane damage, some AMPs and AMP-like polycationic peptides can also cross membranes through transient or pre-existing pores. This has been shown for arginine-rich systems, in which membrane destabilization can nucleate transient hydrophilic pores that permit peptide translocation, and for protamine, a highly arginine-rich antimicrobial peptide that can be electrophoretically driven through bacterial outer-membrane nanopores such as CymA [21]. Such translocation can be influenced by peptide charge and membrane potential, including electrophoretic transport of charged peptides and electroosmotic flow within pores, which may contribute to the intracellular accumulation of some AMP-like molecules.

For example, intracellularly active AMPs can bind nucleic acids, inhibit ribosomal translation, interfere with protein folding, inhibit enzymes, or disrupt essential metabolic pathways; these non-membrane mechanisms are increasingly recognized as important contributors to AMP potency and spectrum of activity [22,23,24]. This non-membrane dimension is supported by recent target-identification work. Wu et al. identified an intracellular target of the antimicrobial peptide EWAMP-R and linked its secondary bactericidal activity to intracellular pathway activation, reinforcing the idea that some AMPs act through combined membrane and intracellular mechanisms [24].

Recent AMP research has also expanded toward biofilm-associated infections. This is particularly important because biofilms are now recognized as central contributors to chronic, recurrent, and device-associated infections, where antibiotic tolerance is often driven by matrix protection, altered metabolic states, persister cells, nutrient gradients, and stress-response adaptation [25]. Biofilms are structured microbial communities embedded in extracellular polymeric substances that protect bacteria from antibiotics, immune attack, nutrient limitation, and environmental stress. This protection contributes to persistent infections and treatment failure, especially in chronic wounds, implanted devices, respiratory infections, and gastric infections [26,27,28,29,30]. AMPs are promising in this context because they can act against planktonic cells, interfere with biofilm formation during early adhesion, disrupt mature biofilms and target persister-like cells, or sensitize biofilm-embedded bacteria to conventional antibiotics [9,31]. Recent antibiofilm AMP studies further support their potential against polymicrobial, oral, wound, and medical-device-associated biofilms, while emphasizing that efficacy should be evaluated in mature and physiologically relevant biofilm models rather than in planktonic assays alone [11,12,32,33]. This point is illustrated by recent studies in clinically relevant pathogens *A. baumannii* [12], *H. pylori* [33], and multidrug-resistant *S. aureus* [11]. The latter study showed that the optimized amphipathic peptide A24 (S9A) displayed activity against multidrug-resistant *S. aureus* and associated biofilms [11]. Mechanistically, A24 (S9A) disrupted the bacterial membrane and increased cell permeability, resulting in rapid killing of S. aureus [11]. Importantly, its activity was not limited to planktonic cells: A24 (S9A) also inhibited early biofilm formation, disrupted mature biofilms, and eliminated persister cells [11]. Although the precise mechanisms responsible for its antibiofilm and anti-persister effects remain incompletely defined, these results suggest that A24 (S9A) may retain its activity against biofilm cells despite physiological adaptations that usually reduce antibiotic susceptibility, including altered metabolism, reduced growth rate, matrix protection, and changes in envelope organization [25].

Despite their promise, relatively few AMPs have reached clinical use. Major barriers include proteolytic degradation, reduced activity under physiological salt or serum conditions, hemolysis, mammalian-cell toxicity, short half-life, poor tissue distribution, immunogenicity concerns, and manufacturing costs [34]. These limitations explain why many recent studies no longer treat AMP discovery and AMP optimization as separate processes. Instead, therapeutic development increasingly integrates rational sequence design, chemical modification, formulation, targeted delivery, surface immobilization, and computational screening to improve stability, selectivity, pharmacokinetics, and antibiofilm performance. Consequently, the current research frontier is not only the discovery of new AMPs but also their optimization through sequence engineering, chemical modification, formulation, surface immobilization, and computational design.

This transition is especially relevant for wound healing and skin-contact applications. Chronic and infected wounds are biologically complex environments characterized by excessive protease activity, inflammatory exudate, impaired tissue repair, polymicrobial colonization, and biofilm formation [30,35,36]. Under these conditions, soluble AMPs may be rapidly diluted, degraded, sequestered by host or matrix components, or neutralized before reaching an effective local concentration. Hydrogel coatings, wound dressings, and topical biomaterial platforms therefore offer an attractive strategy to convert AMPs from short-lived soluble antimicrobials into locally retained bioactive molecules with controlled release, improved stability, reduced systemic exposure, and better integration with tissue-repair processes. This review therefore places AMP engineering within the broader framework of hydrogel-based wound care, antibiofilm biomaterials, and skin-compatible antimicrobial delivery systems.

At the same time, the design of AMPs for these applications is becoming increasingly computationally driven. The challenge is no longer limited to identifying peptides with strong antimicrobial activity, but to discovering and optimizing candidates that remain active in complex wound-like environments, tolerate formulation into biomaterials, display antibiofilm activity, and maintain acceptable cytocompatibility.

A particularly important shift has occurred in the past few years. AMP discovery is moving from organism-by-organism screening toward AI-assisted exploration of enormous peptide sequence spaces. In 2024, Santos-Júnior et al. used machine learning to mine 63,410 metagenomes and 87,920 prokaryotic genomes, generating AMPSphere, a catalog of 863,498 non-redundant candidate AMPs [37]. Similarly, deep-learning-based “molecular de-extinction” has been used to mine more than 10 million peptides from extinct organisms [38]. These studies illustrate a new paradigm in which computational models are used not only to classify known peptides, but also to reveal hidden, cryptic, or entirely novel antimicrobial sequence spaces. This paradigm has expanded further through peptide language models, diffusion models, generative neural networks, and Bayesian optimization strategies that can prioritize candidates according to multiple properties, including antimicrobial potency, toxicity, solubility, stability, safety, synthesizability, and delivery compatibility [39,40,41].

## 2. AMPs as Antimicrobial and Antibiofilm Agents

**Figure 1:**
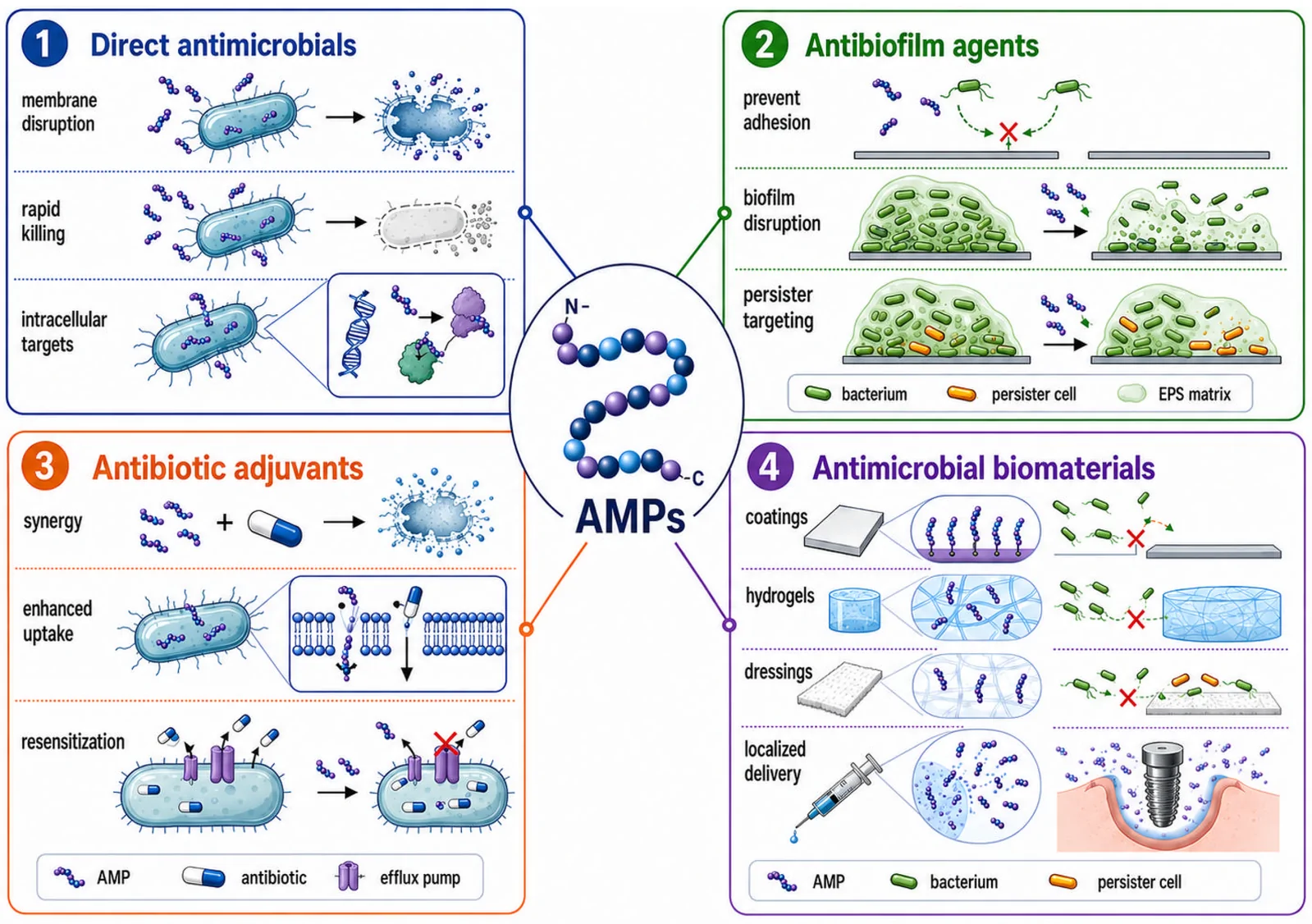
Major roles of antimicrobial peptides in infection control. Schematic summary of the four main applications of antimicrobial peptides (AMPs): direct antimicrobial killing, antibiofilm activity, antibiotic adjuvant effects, and incorporation into antimicrobial biomaterials. AMPs can disrupt bacterial membranes, target intracellular processes, prevent or disrupt biofilms, enhance antibiotic activity, and provide localized antimicrobial protection through coatings, hydrogels, dressings, or delivery systems.

AMPs are currently investigated in four complementary roles: direct antimicrobials, antibiofilm agents, antibiotic adjuvants, and functional components of antimicrobial biomaterials (see Figure 1). These roles are interconnected rather than mutually exclusive, because the same peptide may combine membrane activity, intracellular effects, biofilm disruption, antibiotic potentiation, and local delivery depending on its sequence, formulation, and biological context.

### 2.1. AMPs as Direct Antimicrobial Agents

As direct antimicrobials, AMPs are being explored against Gram-positive and Gram-negative multidrug-resistant pathogens, including *S. aureus*, *Pseudomonas aeruginosa*, *A. baumannii*, *Escherichia coli*, and *Klebsiella pneumoniae*. Their rapid membrane-associated activity and multi-target physicochemical mechanisms may reduce the likelihood of classical single-target resistance.

However, AMPs are not resistance-proof, and resistance propensity should be considered an important design and development parameter. In a systematic experimental evolution study comparing 14 AMPs with 12 antibiotics, Spohn et al. found that antibiotic-adapted *E. coli* lines reached a median resistance increase of approximately 90-fold, whereas AMP-adapted lines showed significantly lower and more heterogeneous resistance. While polymyxin B resistance exceeded 3000-fold, tachyplesin II and R8 showed no detectable resistance, and cecropin P1, indolicidin, PGLA, and pleurocidin remained below threefold on average, highlighting resistance propensity as an important AMP design parameter [14].

These findings indicate that AMPs may reduce, but do not eliminate, the risk of resistance development. This distinction is important because AMP development should include serial-passage assays, cross-resistance analysis, and mechanistic studies rather than relying only on the assumption that membrane-active peptides are intrinsically resistance-proof.

### 2.2. AMPs as Antibiofilm Agents

AMPs are particularly attractive against biofilm-associated infections because their membrane-associated and physicochemical modes of action may allow activity against both metabolically active and slow-growing or persister-like attached bacteria, whereas many conventional antibiotics are less effective against non-dividing biofilm cells. This makes AMPs relevant not only for preventing initial colonization but also for targeting established biofilms and biofilm-associated tolerant subpopulations.

Across recent studies, three common antibiofilm functions emerge: inhibition of early bacterial adhesion, disruption or weakening of established biofilm biomass, and killing or sensitization of tolerant biofilm-associated cells. Rodríguez-Rojas et al. directly tested this concept by exposing stationary-phase/non-dividing *E. coli* and *S. aureus* to cecropin A, LL-37, melittin, pexiganan, and indolicidin. All five AMPs reduced the viability of stationary-phase bacteria, with indolicidin showing the strongest activity against both fast- and slow-replicating cells, whereas conventional antibiotics showed limited killing of non-dividing bacteria under comparable conditions [42]. In mature-biofilm models simulating prosthetic joint infection, Scheper et al. further showed that SAAP-148 eradicated methicillin-resistant *S. aureus* (MRSA) persisters within antibiotic-exposed mature biofilms, supporting the potential of selected AMPs against dormant-like biofilm subpopulations [43]. More recent studies further support this trend: the optimized amphipathic peptide, A24 (S9A) and microbiome-derived Lynronne peptides combine activity against multidrug-resistant pathogens with effects on biofilm growth, adhesion, and mature biofilms [11]. A24 (S9A) was also capable of eliminating 99.9% of persister cells [12] and showed promising results in mouse peritonitis and endometritis models [11].

These studies collectively suggest that the value of AMPs in biofilm-associated infections lies less in a single universal mechanism than in their ability to act across several biofilm stages. However, antibiofilm claims should be interpreted carefully because outcomes depend strongly on the model used, including whether assays measure biomass reduction, viable-cell killing, biofilm prevention, mature-biofilm eradication, or regrowth after treatment.

### 2.3. AMPs as Antibiotic Adjuvants

AMPs can also function as antibiotic adjuvants. By increasing membrane permeability or weakening biofilm structure, AMPs may enhance antibiotic accumulation or restore sensitivity in tolerant or resistant bacterial populations. This adjuvant role is conceptually important because it positions AMPs not only as antibiotic replacements, but also as a tool to extend the useful life of existing antibiotics.

A recent example is GN1, a non-membrane-active peptide that resensitized MRSA *S. aureus* to β-lactam antibiotics and reduced its virulence, illustrating that AMP-like molecules can act as resistance-reversal agents rather than only as membrane-disruptive antimicrobials [44]. A complementary strategy was recently illustrated by a covalent vancomycin–AMP conjugate generated by linking vancomycin to a cysteine-modified MSI-78 peptide through the heterobifunctional crosslinker Sulfo-SMCC [45]. This design combines vancomycin-mediated cell-wall targeting with the membrane-active properties of MSI-78. Compared with vancomycin, Vm–MSI showed an overall 20.18-fold improvement in antimicrobial activity and broadened activity toward vancomycin-resistant *S. aureus* and Gram-negative bacteria, including efficacy in a multidrug-resistant *A. baumannii* lung infection model [45]. Vm–MSI also displayed improved activity against tolerant bacterial states, reducing ciprofloxacin-induced *S. aureus* persister cells by more than 4 logs, compared with approximately 1-log reduction for vancomycin and 3-log reduction for MSI-78 alone [45].

Together, these examples illustrate two distinct adjuvant strategies: peptides can either modulate bacterial physiology to restore antibiotic susceptibility or be chemically integrated with antibiotics to combine targeting and membrane-active functions. In both cases, combination therapy requires careful evaluation because additive, synergistic, indifferent, or antagonistic interactions may depend on peptide concentration, antibiotic class, bacterial species, growth phase, and biofilm state.

### 2.4. AMPs and AMP-Inspired Antimicrobial Biomaterials

Beyond soluble peptide–antibiotic combinations, AMP-based strategies are increasingly being integrated into biomaterials to provide localized and sustained antimicrobial activity. This approach is particularly relevant for infections in which early microbial adhesion and surface colonization initiate biofilm formation, such as wounds, catheters, orthopedic implants, dental materials, and regenerative scaffolds. In these settings, AMP-loaded or AMP-functionalized materials can act as local reservoirs for controlled peptide release or as contact-killing surfaces that maintain antimicrobial activity at the material–tissue or material–microbe interface.

This concept has also been extended to AMP-inspired antimicrobial macromaterials, including cationic homopolypeptide-based nanocoatings. Sequence-defined cationic homopolypeptides, such as poly-L-arginine and poly-ε-lysine, can be assembled with hyaluronic acid to form antimicrobial multilayer coatings [46,47]. Although these systems share key physicochemical mechanisms with AMPs, including electrostatic interaction with bacterial envelopes and membrane perturbation, they should be distinguished from classical AMPs. Classical AMPs are generally discrete peptide sequences with defined residue order, length, and often structure-dependent mechanisms. By contrast, cationic homopolypeptides and synthetic polycationic polymers behave more as antimicrobial macromaterials, with activity mainly governed by charge density, chain architecture, hydrophobicity, and surface presentation. They are therefore better considered AMP-inspired antimicrobial biomaterials rather than conventional AMPs.

For example, poly-L-arginine/hyaluronic acid and poly-ε-lysine/hyaluronic acid coatings have been applied to medical fabrics, wound-dressing materials, urinary catheters, and implant-relevant surfaces, where they reduced bacterial attachment, limited biofilm formation, and provided localized contact-killing activity while maintaining acceptable biocompatibility in vitro and/or in vivo [48,49,50,51,52,53]. In these systems, hyaluronic acid acts as an anionic biopolymer that electrostatically complexes the cationic antimicrobial homopolypeptides, enabling their local concentration, retention, and organization within the multilayer coatings.

By concentrating antimicrobial activity at the surface, such biomaterial strategies may reduce systemic antibiotic exposure, prolong antimicrobial protection, prevent early bacterial adhesion, and inhibit the progression from initial colonization to mature biofilm development on medical surfaces.

## 3. Engineering and Modification Strategies to Improve AMP Properties

AMP engineering aims to improve the therapeutic index by increasing antimicrobial potency and stability while reducing toxicity toward mammalian cells. This requires careful balancing of charge, hydrophobicity, amphipathicity, helicity, solubility, protease resistance, serum stability, and manufacturability. The most common modification strategies include amino acid substitution, D-amino acid incorporation, N-substituted glycin incorporation, cyclization, lipidation, PEGylation, terminal modification, hydrocarbon stapling, hybridization, sequence truncation and metal coordination. These approaches can be broadly grouped into strategies that improve stability, such as D-amino acid substitution, peptoid design, cyclization, PEGylation, and stapling; strategies that enhance membrane interaction or potency, such as lipidation, terminal amidation, hybridization, and metal coordination; and strategies that improve translational feasibility, such as truncation, polymer conjugation, and biomaterial-compatible modification. However, most strategies involve trade-offs, and improvements in potency or stability may be accompanied by changes in toxicity, selectivity, solubility, cost, or biofilm performance.

### 3.1. D-Amino Acid Substitution

D-amino acid substitution is one of the most widely used strategies to increase proteolytic stability. Because many proteases preferentially recognize natural L-amino acid backbones, replacement with D-amino acids can reduce enzymatic degradation and prolong peptide activity [54]. Recent D-amino acid-modified peptides have shown markedly improved protease resistance and preserved antimicrobial activity under physiologically relevant stress conditions. Zhong et al. designed AMP 1003, a linear peptide composed of four repeating units of D-tryptophan, D-arginine, and D-lysine, to overcome the enzymatic instability of the corresponding L-amino acid peptide (WRK)4 [55]. After 6 h exposure to trypsin, chymotrypsin, or pepsin, AMP 1003 retained more than 90% of the intact peptide, whereas (WRK)4 retained less than 10%. Its antimicrobial activity also remained largely unchanged in the presence of digestive proteases, with minimal inhibitory concentration (MIC) values fluctuating only within a twofold range even at the highest protease concentrations tested. In addition, AMP 1003 maintained activity under physiological salt and serum conditions, showed broad-spectrum activity against multidrug-resistant MRSA and *Klebsiella pneumoniae*, and displayed low hemolytic toxicity, supporting D-amino acid conversion as an effective strategy to enhance AMP stability without necessarily compromising antimicrobial performance [55].

Beyond stability, D-amino acid substitution can also influence peptide conformation, membrane interaction, and immune recognition. After D-amino acid substitution, the peptide may show increased resistance to enzymatic degradation, but its secondary structure, amphipathic organization, membrane insertion, and toxicity profile can also change. Partial D-substitution may disrupt helicity or introduce local conformational flexibility, whereas full D-conversion can generate mirror-image peptide architectures that retain activity if the global charge and hydrophobic pattern are preserved. For example, D- and unnatural amino acid substitutions in Pep05 derivatives improved protease resistance but also altered helicity, hydrophobicity, toxicity, and in vivo activity, showing that stereochemical modification must be optimized in a sequence-dependent manner [56].

Although most ribosomally synthesized peptides are composed of L-amino acids, D-amino acids occur naturally in several peptide antibiotics and bioactive peptides. In non-ribosomal peptide antibiotics, such as Gramicidin S, D-amino acids can be incorporated through enzymatic epimerization during biosynthesis. Thus, Gramicidin S is a natural cyclic decapeptide containing D-phenylalanine, making it an early example of a peptide antibiotic in which stereochemistry contributes to structure, stability, and biological activity [57].

### 3.2. Peptoids

Peptoids are peptide mimetics whose backbones are composed of N-substituted glycine residues. Through appropriate side-chain substitutions, they can reproduce several physicochemical and biological properties of conventional peptides. In standard peptides, the backbone amide nitrogen is typically secondary, whereas N-substitution converts it into a tertiary amide lacking an N–H hydrogen-bond donor. Consequently, peptoids cannot participate in backbone hydrogen bonding in the same manner as conventional peptides. This structural feature contributes to their high proteolytic stability because many proteases rely on peptide-backbone recognition and hydrogen-bonding interactions for substrate binding and cleavage. However, the absence of backbone hydrogen-bond donors also alters their conformational behavior. Because conventional α-helices and β-sheets are strongly stabilized by backbone hydrogen bonding, peptoids generally adopt different secondary structures and conformational preferences. It is therefore important to determine whether the biological activity of the parent peptide depends on its native secondary structure before converting it into a peptoid analogue.

Peptoids have shown promising results in their antimicrobial and antibiofilm activities. For instance, Wardell et al. designed a lipo-peptoid called TM18, whose MIC was shown to range from 1.6 to 12.5 µg/mL, depending on the broth (Mueller Hinton broth or tissue culture medium supplemented with fetal bovine serum and glucose) and the strain (*S. aureus* LAC USA300 or *P. aeruginosa* LESB58) [58]. They tested this peptide against biofilms of both strains, individually and in dual-species biofilm. While TM18 had a limited effect on *P. aeruginosa*, it had a pronounced effect on *S. aureus* and dual-species biofilms, with more than 50% reduction in mass compared to PBS. TM18 has also been shown to have a synergistic effect with meropenem, an antibiotic often used in *P. aeruginosa* infections. While reducing the abscess size in mouse models, combining TM18 with meropenem also significantly decreased the number of CFUs of both bacterial species.

Peptoids also have the advantage in terms of scalability. While AMPs mainly rely on solid-phase peptide synthesis (SPPS), a large variety of techniques already exist for peptoid synthesis to avoid reliance on SPPS, which is costly, time-consuming and difficult to scale up. In their review, Xie et al. summarized polymer-chemistry approaches that could become useful for peptoid synthesis [59]. N-substituted N-carboxyanhydride (NNCA), N-substituted N-thiocarboxyanhydride (NNTA) and N-phenoxycarbonyl N-substituted glycines (NNPC) are three relevant monomers that lead to a wide range of reactions yielding different molecular topologies, such as cyclic or linear peptoids, depending on the initiator and catalyst used to perform the polymerization.

Together, D-amino acid substitution and peptoid design illustrate two related but distinct approaches to protease resistance. D-amino acid substitution preserves the peptide backbone but changes stereochemistry, whereas peptoids modify the backbone itself. Both can improve stability, but both also require careful reassessment of antimicrobial activity, secondary structure, membrane interaction, antibiofilm efficacy, and toxicity.

### 3.3. Topological Engineering: Cyclization, Branching, and Multivalent Architecture

Cyclization can improve peptide stability by constraining conformation and reducing susceptibility to exopeptidases or endopeptidases. Recent studies support this context-dependent benefit. Mitra et al. showed that the cyclic peptides CE-03 and CE-05 retained antibacterial activity after elastase exposure, whereas the linear helical AMP E2-35 lost activity after proteolysis [60]. Similarly, Pei et al. reported that the cyclic peptide C-LR18, generated from LR18 by end-to-end cyclization via disulfide bonds, showed improved activity retention after exposure to trypsin, carboxypeptidase, papain, serum, and artificial gut fluids, together with longer plasma and in vivo half-life than the linear parent peptide [61]. However, these examples should not be generalized to all cyclic AMPs: the protective effect of cyclization is peptide-, topology-, and protease-dependent, and stability must therefore be validated under the biological conditions relevant to the intended application.

Cyclization can be achieved through head-to-tail linkage, disulfide bridges, lactam bridges, thioether bonds, or other chemical linkages. However, cyclization can also reduce flexibility or disrupt the active conformation, making structure–activity optimization essential.

Gramicidin S provides an important example of cyclic peptide scaffold optimization. The parent molecule is a natural cyclic decapeptide containing D-phenylalanine and ornithine, but its therapeutic use is limited by hemolytic toxicity and a narrow therapeutic window. Kalyvas et al. redesigned this scaffold through targeted residue substitutions that tuned cationicity and hydrophobicity, including incorporation of D-Arg, Trp, and non-proteinogenic tert-leucine residues. These modifications improved activity against Gram-negative ESKAPE pathogens while reducing hemolytic and mammalian-cell toxicity, illustrating how historical cyclic AMP scaffolds can be re-engineered into more drug-like candidates [57].

Branching creates a multivalent three-dimensional architecture with several peptide arms emerging from one core. The goal is not to constrain the peptide into a ring, as in cyclization, but to improve multivalency, spatial presentation, local charge density, and membrane interaction. Lebaudy et al. synthesized branched poly-L-arginine peptides belonging to the multiple antigenic peptide family and compared them with linear poly-L-arginine analogues containing the same number of arginine residues. The branched architecture improved antibacterial activity compared with the corresponding linear peptides, showing that peptide topology can influence antimicrobial function independently of amino acid composition. Molecular dynamics simulations further suggested that branched poly-L-arginine structures interact more strongly with bacterial membrane models and induce greater membrane distortion than linear analogues. Interestingly, increasing the number of arms or arginine residues did not automatically increase antibacterial activity, indicating that branching must be optimized rather than simply maximized.

Cyclization and branching are strategies for modifying peptide topology, often to improve conformational stability, protease resistance, multivalency, and spatial presentation. However, these topological modifications could affect potency, hemolysis, cytotoxicity, and selectivity.

### 3.4. Lipidation

Lipidation involves conjugation of fatty acid chains or lipid groups to increase hydrophobicity and membrane affinity. This conjugation is often achieved through N-terminal acylation, but several strategies can be used, such as click chemistry, thioether, or disulfide-bond formation [62]. This strategy can significantly improve antibacterial potency, especially against membrane targets, and may enhance self-assembly or tissue retention. Sometimes, a glycine linker is added between the AMP and the lipid chain. Chen et al. tried to understand the effect of the length of this linker on the antimicrobial activity of octanoyl lipopeptides. They found that a one-glycine linker increased micelle stability formed by the modified peptide, leading to weak interactions with the cell membrane, resulting in lower antimicrobial activity. However, it was also shown that longer linkers reduced micelle stability and consequently increased antimicrobial activity [63]. Another important aspect of this modification is the length of the added lipid. Squitieri et al. compared two lipidated Trem-4 peptides, one with caprylic (C8) and the other with myristic (C14) fatty acid chains. Both lipidated peptides were shown to have a significantly improved interaction with the membranes, while showing different aggregation and selectivity profiles. While Cap-T4 showed consistent results, Myr-T4 had enhanced activity against some strains [64]. However, increased hydrophobicity can also increase hemolysis and cytotoxicity. As shown by Squitieri et al., Myr-T4 showed higher cytotoxicity than Cap-T4 in vitro [64]. Lipid chain length, attachment site, linker size, peptide sequence, and net charge must therefore be optimized to maintain bacterial selectivity [65].

Thus, lipidation is one of the most effective strategies for increasing membrane affinity and antimicrobial potency, but it is also one of the strategies most likely to increase mammalian-cell toxicity if hydrophobicity is not carefully controlled. For biofilm-associated infections, lipidation may be particularly useful when stronger interaction with bacterial membranes or biofilm matrices is required, but its translational value depends on maintaining an acceptable selectivity window.

### 3.5. PEGylation and Polymer Conjugation

PEGylation and polymer conjugation are used to improve solubility, circulation time, and pharmacokinetics. These modifications may protect peptides from proteases and reduce renal clearance. Fontanot et al. demonstrated that the PEGylated peptide SET-M33L-PEG showed lower MIC and MBC values and improved antibiofilm activity compared with the non-PEGylated peptide against MDR *P. aeruginosa* [66]. Similarly, Cui et al. showed that the biological impact of PEG conjugation depends strongly on polymer architecture: comb-like PEG–AMP conjugates displayed higher helicity, solubility, antimicrobial activity, and proteolytic stability than star-shaped analogues at comparable molecular weight and peptide content [67]. Importantly, the same study showed that PEG side-chain length and backbone architecture modulate the balance between peptide exposure, proteolytic shielding, and antimicrobial potency, highlighting that PEGylation can be optimized rather than applied uniformly [67].

However, polymer shielding can also reduce antimicrobial activity by masking cationic or amphipathic regions required for bacterial membrane interaction. For AMP development, PEGylation should therefore be considered as a tunable pharmacological strategy rather than as a universal improvement.

### 3.6. Terminal Amidation and Sequence Optimization

C-terminal amidation is common among natural and synthetic AMPs and can increase net positive charge, improve helicity, enhance membrane binding, and protect against carboxypeptidase degradation. Recent evidence further supports the functional relevance of this modification. Van Wyk et al. showed that carboxy-amidation of the scorpion-derived peptide AamAP1-Lys generated AamAP1-Lys-NH_2_, which displayed greater conformational flexibility, faster bactericidal activity against Gram-negative bacteria, improved membrane permeabilization and penetration, enhanced antibiofilm activity, increased selectivity, and improved protection in a *Galleria mellonella* burn-wound infection model [68].

Beyond terminal modification, rational sequence optimization can be used to fine-tune AMP charge, amphipathicity, hydrophobic moment, helicity, and toxicity. N-terminal acetylation, residue substitution, sequence reordering, and modulation of lysine/arginine content may improve stability or reduce cytotoxicity while preserving antimicrobial function. For example, van der Walt et al. demonstrated that lysine-to-arginine enrichment of the scorpion venom-derived peptide Opis16a enhanced Gram-negative membrane disruption, improved antimicrobial selectivity, increased serum stability, and improved in vivo efficacy in a burn-wound infection model [69].

The incorporation of unnatural amino acids represents another strategy for expanding AMP chemical diversity and tuning peptide activity. By introducing residues with enhanced hydrophobic side chains, Marata et al. designed and synthesized hybrid peptides with improved antimicrobial properties by incorporating the unnatural amino acid D-Tic, together with a palmitoylated lysine-based residue, Lys(Glu-Pal), to tune peptide hydrophobicity and antimicrobial activity [70]. In particular, peptide 6e showed potent antibacterial activity, with MIC values of 2 µg/mL against *E. coli*, 0.2 µg/mL against *Bacillus subtilis*, and 0.3 µg/mL against *Bacillus megaterium* and *Streptococcus pyogenes*, values that were comparable to or better than those obtained with ampicillin under the same assay conditions [70]. The same peptide also displayed antifungal activity, including MIC values of 2 µg/mL against *Candida albicans* and 7.5–8 µg/mL against *Aspergillus niger*, *Aspergillus oryzae*, and *Rhizopus* spp., comparable to fluconazole in this experimental setting [70]. Mechanistically, molecular docking and 100 ns molecular dynamics simulations suggested stable interactions of peptide 6e with the transglycosylase domain of *E. coli* PBP1b, while the antifungal activity was proposed to involve membrane-disruptive effects linked to the hydrophobic side-chain design. Together with terminal amidation and lysine/arginine-based sequence optimization, these findings illustrate how residue-level engineering can tune AMP hydrophobicity, membrane interaction, target binding, biological stability, and antimicrobial performance.

### 3.7. Hydrocarbon Stapling

Hydrocarbon stapling is a conformational constraint strategy mainly applied to α-helical AMPs [71,72]. Chemically, it is usually introduced by replacing two residues located on the same face of the helix with α,α-disubstituted non-natural amino acids bearing terminal alkene side chains. After SPPS, these alkene-bearing residues are covalently connected by ring-closing metathesis, typically using a ruthenium-based Grubbs catalyst, generating an all-hydrocarbon bridge that stabilizes the helical conformation [72]. For AMPs, this constraint can improve helicity, protease resistance, membrane interaction, antibacterial activity, and sometimes antiendotoxin activity, but the outcome depends strongly on staple position, peptide sequence, amphipathic organization, charge distribution, and hydrophobicity [71,72].

Recent examples illustrate this potential. Yang et al. generated hydrocarbon-stapled analogues of the frog-derived host-defense peptide Ocellatin-3N/Oce-3N-0 and showed that selected stapled analogues displayed improved antimicrobial activity and protease resistance compared with the parent peptide, with Oce-3N-5 identified as a promising lead [73]. Mahto et al. similarly developed a hydrocarbon-stapled analogue of temporin-L, a short frog-derived AMP, and reported enhanced antibacterial and antiendotoxin activity together with improved protease stability [74]. These studies support hydrocarbon stapling as a useful strategy for optimizing helical AMPs. However, incorrectly positioned staples can disrupt the amphipathic helix, increase hydrophobicity, reduce bacterial selectivity, or promote nonspecific interactions with mammalian membranes. Compared with D-amino acid substitution or cyclization, hydrocarbon stapling provides more precise stabilization of α-helical conformations, but it is chemically more complex and may increase hydrophobicity and synthesis cost. Therefore, stapling is most useful when helicity is directly linked to activity, whereas it may be less suitable for peptides whose function depends on conformational flexibility or non-helical structures.

### 3.8. Hybridization and Truncation

Hybrid peptides combine motifs from two or more parent AMPs to merge desirable properties such as potency, selectivity, antibiofilm activity, lipid binding, pathogen targeting, or immunomodulatory capacity. Sequence truncation can also reduce synthesis cost and remove toxic or unstable regions while preserving the minimal active core. Recent studies illustrate how these strategies are usually applied through iterative design rather than simple sequence fusion or shortening. Wongchai et al. designed the hybrid antibiofilm peptide BiF2_5K7K by deriving a conserved sequence from 18 α-helical antibiofilm peptides, hybridizing it with the KILRR lipid-binding motif, and further optimizing the sequence by lysine substitution. The resulting peptide showed dose-dependent antibiofilm activity against biofilm-forming *Staphylococcus epidermidis*, inhibited biofilm formation at subinhibitory concentrations by affecting extracellular polysaccharide production and quorum-sensing gene expression, retained activity after human serum exposure, and showed low hemolysis and mammalian-cell toxicity [75].

Similarly, Wu et al. used rational design strategies, including truncation, amino acid replacement, and “heterozygosity”, to generate Abhisin-like peptides with α-helical, cationic, and broad-spectrum antimicrobial properties. The best candidate, AB7, showed potent antimicrobial activity, membrane-targeting and DNA-binding activity, antibiofilm effects and therapeutic efficacy in a murine methicillin-resistant *S. aureus* (MRSA)-infected wound model [76]. Truncation and analogue optimization can also preserve antibacterial activity while improving antibiofilm performance. For example, Akhash et al. designed mKLK from a D-form amidated sapecin B-derived peptide by targeted residue replacement and insertion; mKLK retained antibacterial activity and serum stability but showed improved inhibition, eradication, and dispersal of methicillin-resistant and methicillin-sensitive *S. aureus* biofilms, including activity in a murine catheter-associated biofilm model [77]. These examples show that hybridization and truncation are not only cost-reduction strategies, but also practical routes to tune amphipathicity, charge distribution, stability, antibiofilm activity, toxicity, and application-specific performance. However, motif fusion or sequence shortening can also disrupt folding, amphipathic organization, target binding, or selectivity, and therefore require systematic antimicrobial, antibiofilm, stability, and toxicity screening. Compared with stapling, PEGylation, or lipidation, hybridization and truncation are simpler sequence-based strategies, but their effects are less predictable because they can alter charge, amphipathicity, folding, antibiofilm activity, and toxicity.

### 3.9. Metal Coordination

Metal coordination is an emerging strategy for modulating the structure and function of AMPs. In this approach, metal-binding residues within the peptide sequence, particularly histidine, cysteine, aspartate, glutamate, or terminal amino groups, interact with transition metal ions such as Cu(II), Zn(II), Ni(II), or Fe(III). This interaction can act as a conformational switch, stabilizing or reshaping the peptide structure, modifying local charge distribution, and altering membrane affinity, thereby potentially affecting the peptide’s antimicrobial activity [78]. The effect is highly metal- and sequence-dependent. Miller et al. recently demonstrated this using PvHCt, a 23-amino-acid His-rich peptide derived from shrimp. They showed that while Cu(II) coordination induced significant structural changes by promoting alpha-helix formation and by creating a strong bend in the peptide backbone, Zn(II) induced only minor structural changes to the peptide, leading to low antimicrobial activity [79].

This technique can also be used to improve selectivity and thus reduce nonspecific cytotoxicity of the antimicrobial peptide. In this case, the ion is not used to give the peptide a given conformation but serves to target the bacterial outer membrane. In their study, Song et al. used Zn(II) to direct their antimicrobial peptoid towards phosphate groups of the lipopolysaccharides and lipoteichoic acids on the bacterial membrane. Upon binding to a phosphate group, the antimicrobial peptide could disrupt the membrane. This approach produced promising results, as they were able to achieve a greater than 10-fold improvement in bacterial membrane selectivity [80].

Finally, Liu et al. developed antimicrobial metallohelices whose antimicrobial activity against Gram-positive bacteria could be tuned by irradiation. This is a promising approach for addressing delivery and systemic toxicity concerns [81]. Irradiation resulted in an 8-fold reduction in MIC against Gram-positive bacteria. Notably, the metallohelices exhibited reversible isomerization. Upon irradiation at 455 nm, the helices returned to their low-activity isomeric state, with only minimal degradation observed after both transformations [81].

Using metals to modulate the antimicrobial activity of AMPs is a promising field. With advanced bioinorganic chemistry, metal coordination expands the possibilities for improving antimicrobial activity, selectivity, and tunability. However, it is important to bear in mind that the optimal metal ion is peptide-dependent; the strategy requires peptides containing suitable metal-binding residues. Although metal coordination is promising, it should be approached cautiously because host metal homeostasis is tightly regulated. Excess or weakly bound metals may promote ROS generation, oxidative damage, and metal-dependent cell-death pathways such as cuproptosis or ferroptosis [82,83]. Therefore, metal–AMP complexes should be evaluated for metal release, redox activity, ROS generation, and host–cell toxicity.

Taken together, these modification strategies offer distinct advantages but also carry specific risks and limitations, and should therefore not be viewed as interchangeable solutions (Table 1). D-amino acid substitution and peptoid design are particularly useful for improving protease resistance; cyclization and stapling are most relevant when conformational stabilization is required; lipidation and terminal amidation can enhance membrane interaction and potency but may narrow the toxicity window; PEGylation and polymer conjugation can improve pharmacokinetics or stability but may shield the active peptide surface; hybridization and truncation allow sequence-level optimization but require careful functional screening; and metal coordination introduces tunability but adds concerns related to metal release and host toxicity. Therefore, the most promising AMP candidates for biofilm-associated infections will likely result from sequence-specific combinations of these strategies, guided by antimicrobial potency, antibiofilm activity, stability, toxicity, manufacturability, and delivery requirements.

## 4. Biomaterial Immobilization and Delivery Systems

For biofilm-associated infections, AMP efficacy depends not only on peptide sequence but also on whether the peptide can be retained at the infected interface, protected from degradation or dilution, and presented at concentrations sufficient to prevent bacterial adhesion or disrupt biofilms. This is particularly relevant for wounds, catheters, implants, and other medical surfaces, where local delivery or surface immobilization can convert short-lived soluble AMPs into sustained antimicrobial reservoirs or contact-killing interfaces. Therefore, this section focuses on biomaterial approaches that directly improve AMP stability, localization, antibiofilm activity, and translational feasibility.

### 4.1. Surface Immobilization and AMP-Functionalized Interfaces

Soluble AMPs often lose activity in biologically complex environments because they can be diluted, degraded by proteases, sequestered by host or matrix components, or reach mammalian cells at toxic concentrations. Surface immobilization addresses these limitations by localizing peptide activity at infection-prone interfaces, such as wounds, catheters, orthopedic implants, dental materials, and regenerative scaffolds. In this context, immobilized AMPs are not discussed as general biomaterial coatings, but as engineering strategies that maintain antimicrobial activity where biofilms initiate: the material–microbe interface.

A series of studies has developed amphiphilic Pluronic F127-based hydrogels, hydrogel particles, and surface coatings in which AMPs are covalently attached to the material, commonly through carbodiimide-mediated EDC/NHS coupling between carboxyl groups on the material and primary amines on the peptide. One representative AMP used in these systems is the synthetic PRELP-derived peptide RRP9W4N, with the sequence RRPRPRPRPWWWW-NH_2_. Atefyekta et al. covalently immobilized RRP9W4N onto amphiphilic, ordered mesoporous Pluronic F127 hydrogels, generating contact-killing surfaces with broad activity against *S. epidermidis*, *S. aureus*, *P. aeruginosa*, MRSA, and multidrug-resistant *E. coli*. This approach improved peptide stability in human serum and showed no detectable toxicity toward human fibroblasts under the tested conditions [84]. Related work adapted AMP-functionalized hydrogel particles as multifunctional coatings for PDMS elastomers, creating stable contact-killing surfaces relevant to medical-device applications [85]. AMP-functionalized hydrogel particles have also been combined with conventional antibiotics. RRP9W4N-functionalized particles retained synergistic or additive effects with oxacillin and vancomycin against *S. aureus* and MRSA, including a 64-fold reduction in oxacillin MIC against MRSA when the AMP was immobilized, compared with a 512-fold reduction for the soluble AMP combination [86].

Together, these studies show that covalent immobilization can transform soluble AMPs into localized, non-leaching, contact-killing surfaces with improved serum stability and reduced systemic exposure. However, immobilization can also restrict peptide mobility, alter orientation, reduce access to intracellular targets, and change activity across bacterial species. Therefore, linker chemistry, surface density, spacer length, peptide orientation, and material architecture must be optimized to preserve antimicrobial and antibiofilm function.

**Figure 2:**
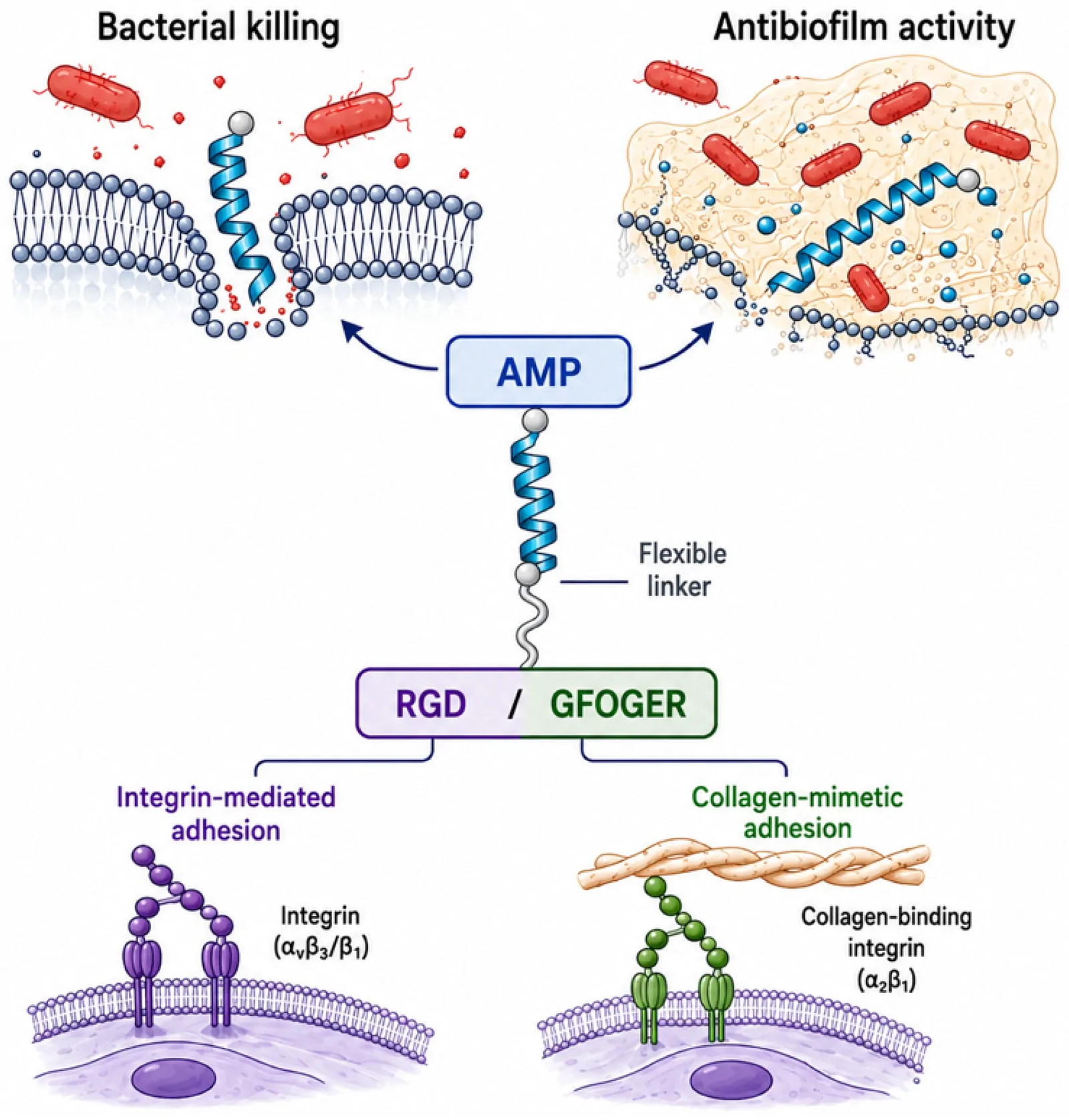
Modular AMP–adhesion motif design for controlled antimicrobial localization and host–cell integration. Schematic showing an AMP domain linked to an Arg-Gly-Asp motif (RGD) or a synthetic, triple-helical collagen-mimetic motif (Gly-Phe-Hyp-Gly-Glu-Arg) (GFOGER) to combine antibacterial/antibiofilm activity with host–cell adhesion. In this design, the AMP domain is intended to reduce early bacterial attachment and surface colonization, thereby preventing the establishment and maturation of bacterial biofilms on the material surface. The linker is a critical design element because it spatially separates the AMP domain from the adhesive motif, helping to preserve antimicrobial activity while keeping the RGD or GFOGER motif accessible for integrin binding.

One approach to improve local retention and tissue compatibility is to combine antimicrobial activity with cell-adhesive motifs (Figure 2). Arg-Gly-Asp (RGD) is an integrin-binding motif widely used to promote mammalian-cell adhesion on biomaterials. Indeed, highly antifouling or highly bactericidal materials may reduce bacterial attachment, but they can also impair adhesion, spreading, and integration of fibroblasts, osteoblasts, endothelial cells, or epithelial cells. RGD-containing designs therefore prevent bacterial colonization while maintaining a surface that host cells can recognize and repopulate [87,88,89,90].

In this strategy, the AMP and adhesive domains should ideally be spatially separated to preserve both bacterial killing and antibiofilm activity, whereas the RGD motif supports integrin-mediated host–cell adhesion. A flexible linker is therefore critical, as it reduces steric interference, keeps the RGD motif accessible, and helps prevent disruption of AMP amphipathicity or unwanted interaction with mammalian membranes [87].

This dual-function concept has been demonstrated in antimicrobial coatings functionalized with both AMPs and RGD peptides, which aim to reduce biofilm formation while supporting tissue integration [87]. Related RGD-modified implant and bone-regeneration materials further show how cell-adhesive motifs can promote osteoblast adhesion and bone formation while antimicrobial components limit bacterial colonization [88,89]. Thus, successful AMP-based coatings must balance bacterial killing with host–cell compatibility and tissue integration [91].

GFOGER is a collagen-derived cell-adhesive motif in which hydroxyproline-containing collagen-like sequences engage collagen-binding integrins, particularly α2β1 and α1β1 [92]. In biomaterial design, GFOGER provides a collagen-mimetic alternative to RGD and can promote osteoblast adhesion, focal adhesion signaling, osteogenic differentiation, and matrix mineralization when presented in an appropriate triple-helical context [93]. When combined with AMPs in modular biomaterials, GFOGER can provide host–cell-adhesive and tissue-integrative cues, while the AMP domain provides antibacterial or antibiofilm activity. This principle was recently illustrated in 3D-printed titanium implants, where a multifunctional peptide combining a titanium-binding domain, KR-12, and GFOGER enabled surface anchoring, antibacterial activity, and bone-related cell responses [94].

For AMP development, the advantage of adding an RGD, GFOGER, or other matrix-binding module is not simply increased “stickiness,” but controlled biological localization. However, these designs require careful optimization because adhesive motifs may alter peptide orientation, increase peptide length and synthesis cost, affect integrin-dependent cell responses, or reduce antimicrobial selectivity if the AMP domain becomes too close to mammalian membranes. Therefore, linker length, motif position, peptide density, orientation, bacterial killing, biofilm inhibition, hemolysis, cytocompatibility, and cell adhesion should be evaluated together.

### 4.2. Nanocarrier-Based Delivery Systems for Antimicrobial Peptides

Despite their strong antimicrobial potential, many antimicrobial peptides (AMPs) show limited performance in biologically complex environments. In serum, wound exudate, mucus, or infected tissue, soluble AMPs may be diluted, sequestered by proteins or extracellular matrix components, degraded by proteases, or reach mammalian cells at concentrations associated with cytotoxicity. These limitations have encouraged the development of nanocarrier-based delivery systems designed to protect AMPs, increase their local retention, and control their release at infected sites. In this context, the carrier is not simply a passive excipient but a functional component that can determine whether an AMP remains active under clinically relevant conditions.

Biopolymer-based nanocarriers provide one promising approach for local AMP delivery. For example, PA-13, an antimicrobial peptide with activity against *P. aeruginosa*, was encapsulated in a chitosan–dextran sulfate nano-network through electrostatic interactions, generating a moldable formulation suitable for topical or injectable administration. This system protected PA-13 from proteolytic degradation and preserved anti-*P. aeruginosa* activity after protease exposure, whereas the free peptide lost activity under the same conditions. Importantly, the nano-network formulation retained efficacy both in vitro and in an ex vivo porcine skin infection model, supporting its relevance for infected wound applications [95]. Such systems are particularly attractive for chronic wounds, where local protease activity and biofilm-associated barriers can rapidly compromise soluble antimicrobial agents.

Hydrogel-based AMP depots have also been investigated as wound-compatible delivery platforms. Encapsulation of the human cathelicidin LL-37 within keratin hydrogels illustrates this strategy. The keratin matrix provides a soft and biocompatible environment suitable for wound applications while enabling localized peptide delivery [96]. This is relevant because LL-37 is not only an antimicrobial peptide but also a host-defense peptide involved in inflammation, angiogenesis, cell migration, and tissue repair. Delivery matrices may therefore support both antimicrobial and regenerative functions by maintaining the peptide within the wound bed for a longer period than would be possible with direct soluble administration.

More advanced nanocarrier systems are being designed to combine antimicrobial activity with wound-healing functions. Recent hydrogel systems containing peptide-based nanocomplexes integrate antibacterial nanoparticles, photothermal effects, and pro-healing peptides within a single local treatment platform [97]. These multifunctional systems reflect a broader shift in AMP development: rather than treating AMPs as short-lived soluble antibiotics, current strategies increasingly aim to engineer spatially controlled, sustained, and infection-responsive local therapies. This is particularly relevant for biofilm-associated infections, where bacterial tolerance, extracellular matrix barriers, and impaired tissue repair occur simultaneously.

Related local-delivery approaches using peptide-like or polycationic antimicrobial biomaterials further support the importance of controlled presentation at infected interfaces. Polyanionic hydrogels loaded with polyarginine have been developed as reservoirs for polycationic antibiotic substitutes, providing prolonged antibacterial activity through gradual release [98]. Subsequent systems combined polyarginine-mediated antimicrobial activity with immunomodulatory and miRNA-delivery functions within hyaluronic-acid hydrogels, linking infection control with inflammation modulation and wound-repair biology [99]. In addition, branched polyarginine architectures have shown improved antibacterial performance compared with linear analogues containing the same number of arginine residues, highlighting the importance of peptide architecture and molecular presentation in antimicrobial biomaterial design [19]. Although these examples are not classical AMP nanocarriers, they reinforce the same translational principle: antimicrobial activity is often more effective when localized, sustained, and integrated into the material environment.

Overall, nanocarrier delivery systems can improve AMPs by protecting them from proteolysis, increasing local retention, reducing systemic exposure, and enabling sustained release at infected sites. Experimental studies show that formulation can determine whether a peptide remains active under biologically relevant conditions. For instance, free PA-13 lost activity after protease exposure, whereas nano-network-formulated PA-13 retained anti-*P. aeruginosa* activity [95]. These findings support the view that delivery engineering is a central component of AMP development, especially for infected wounds, biofilms, and localized *P. aeruginosa* infections.

However, nanocarrier systems also introduce important challenges. Encapsulation can reduce immediate peptide availability, release kinetics may be difficult to tune, and carrier–peptide interactions can alter antimicrobial activity. Formulation also increases manufacturing, scale-up, sterilization, and quality-control complexity. From a regulatory perspective, peptide-loaded nanocarriers, hydrogels, or wound dressings may need to be evaluated as combined therapeutic systems rather than as peptides alone, requiring evidence on carrier biocompatibility, degradation, peptide release, local toxicity, reproducibility, and antimicrobial performance under clinically relevant conditions. A practical solution is to design delivery systems with translation in mind from the beginning: using simple and clinically familiar materials when possible, defining whether the intended product is a topical gel, injectable depot, wound dressing, or local antibiofilm treatment, quantifying peptide loading and release under infection-relevant conditions, and comparing free and formulated peptide side by side. In this way, nanocarriers can help move AMPs from promising in vitro molecules toward realistic local therapies.

## 5. AI-Guided AMP Discovery and Design

**Figure 3:**
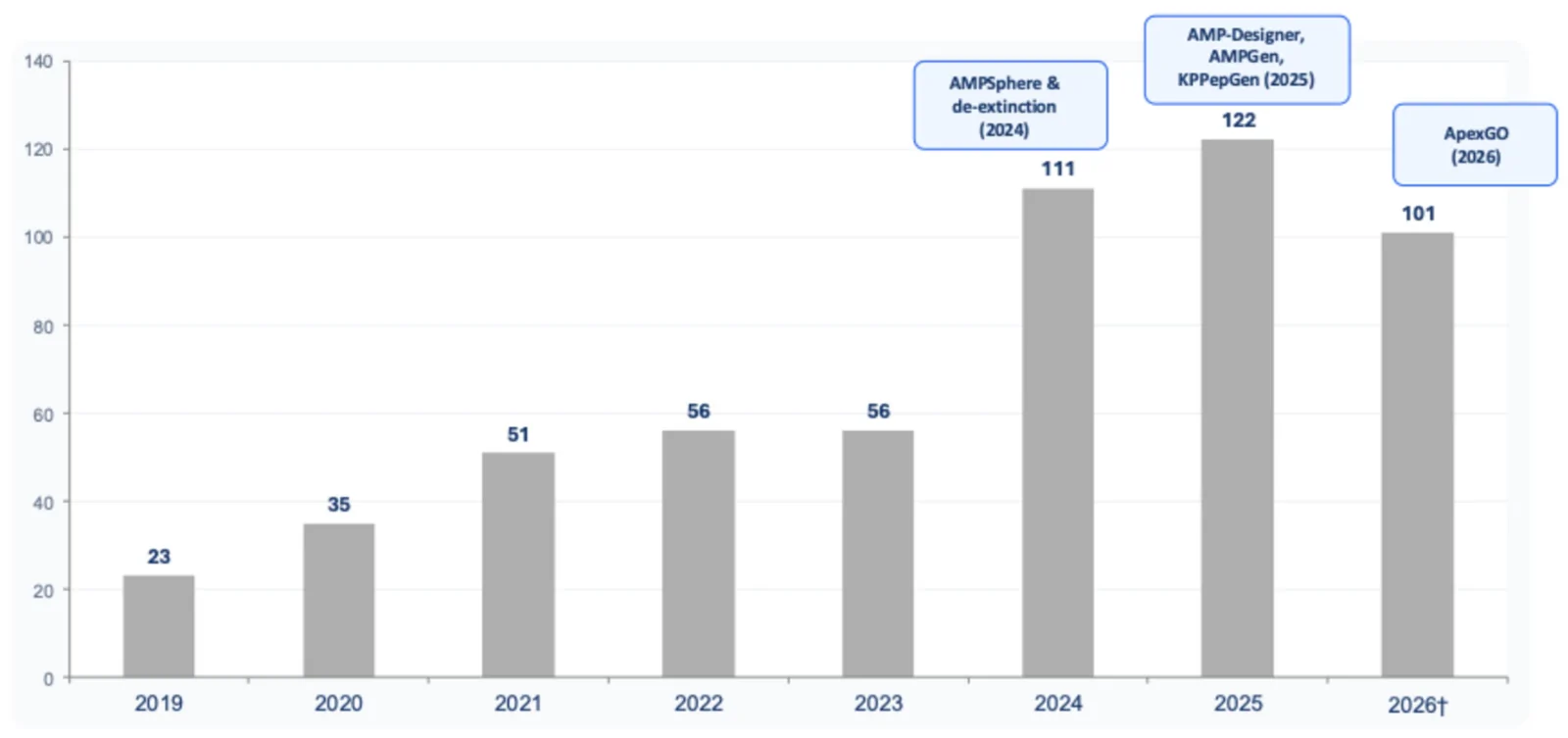
Growth of AI-driven AMP research (2019–2026). Data were retrieved from Web of Science in August 2026 using the search query: TS = (“antimicrobial peptide*”) AND TS = (“machine learning”). Key milestones annotated in the figure include AMPSphere [37], molecular de-extinction [38], AMP-Designer [100], AMPGen [101], KPPepGen [102]; and ApexGO [103]. †: The 2026 value reflects partial-year data from January to August 2026.

Artificial intelligence (AI) is increasingly transforming AMP research by enabling systematic exploration of peptide sequence spaces that are far larger than can be experimentally screened. Here, AI is used as the broad umbrella term encompassing computational approaches designed to perform tasks that typically require human intelligence. Machine learning (ML) represents a subset of AI in which algorithms learn patterns from data, while deep learning (DL) is a subset of ML based on multilayer neural networks. Building on these approaches, generative models, including variational autoencoders, generative adversarial networks, diffusion models, and large language models, can learn sequence distributions and generate novel peptide candidates. The growing importance of these computational approaches is reflected by the rapid increase in AI-driven AMP publications between 2019 and 2026 (Figure 3).

**Figure 4:**
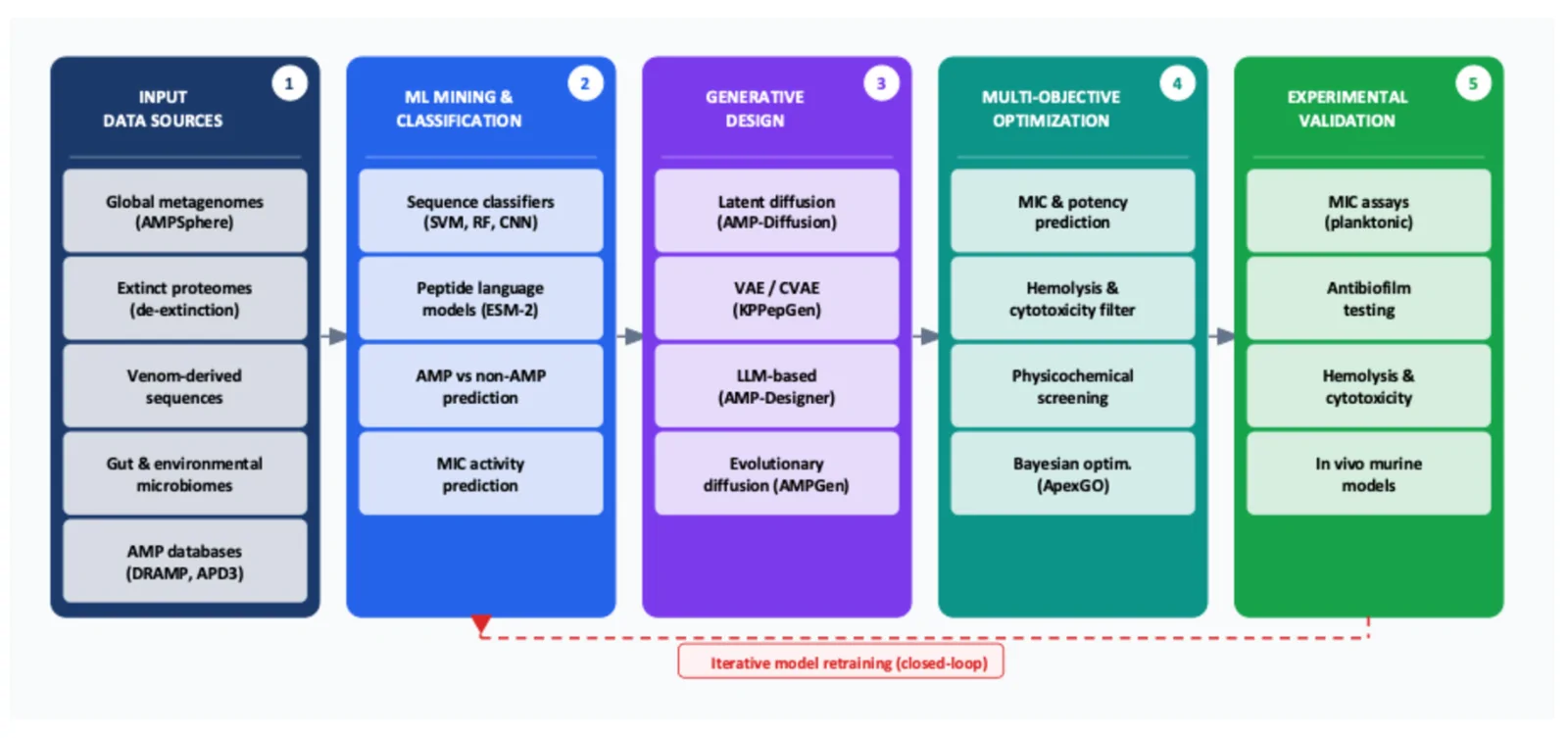
Schematic of the AI-guided AMP discovery pipeline. The pipeline summarizes how AMP datasets are curated and used for model training, prediction, generative design, candidate prioritization, synthesis, and experimental validation. Experimental validation results are fed back into the model through a closed-loop process to iteratively improve candidate quality. Abbreviations: SVM, support vector machine; RF, random forest; CNN, convolutional neural network; ESM-2, Evolutionary Scale Modeling; VAE, variational autoencoder; CVAE, conditional VAE; LLM, large language model; MIC, minimum inhibitory concentration.

This development represents a major shift from traditional AMP discovery, which relied primarly on isolation from natural sources, database mining, or rational modification of known peptides. ML and DL methods now extend these approaches by supporting sequence classification, activity and toxicity prediction, peptide mining, generative design, and multi-objective optimization. Together, these applications can be organized into an integrated AI-guided AMP discovery pipeline that progresses from dataset curation and model training to candidate generation, experimental validation, and iterative model refinement (Figure 4). Importantly, these approaches contribute at different stages of AMP development: mining expands the accessible sequence space, generative models create new candidates, and multi-objective frameworks balance antimicrobial activity with toxicity, stability, and biofilm-relevant properties. Together, they reflect a shift from peptide discovery toward translationally oriented optimization.

### 5.1. Machine-Learning-Based Mining of Biological and Metagenomic Datasets for AMP Discovery

The AMPSphere study is a landmark example of global ML-based AMP mining. Santos-Júnior et al. used machine learning to predict AMP candidates across tens of thousands of metagenomes and nearly 90,000 prokaryotic genomes, generating 863,498 non-redundant candidates [37]. This work demonstrated that global microbiomes contain a vast reservoir of peptide-like antimicrobial candidates, many of which show limited similarity to previously known AMP databases. Among 100 synthesized candidates, 79 showed measurable activity and 63 displayed in vitro antimicrobial activity against clinically significant ESKAPEE pathogens, with leading candidates also showing anti-infective activity in a preclinical animal model, supporting the translational value of large-scale metagenomic mining [37]. Microbiome-derived peptide discovery is particularly attractive because host-associated and environmental microbial communities have evolved under intense interspecies competition, which may generate diverse antimicrobial or competitive molecules.

This mining strategy is now expanding beyond the human gut microbiome. For example, metagenomics and machine learning have been applied to environmental sludge microbiomes, predicting more than 16 million candidate AMPs, while the SEGMA framework mined extremophile metagenome-assembled genomes from environments including glaciers, hot springs, deep-sea sediments, and identified 3298 candidate “extremocins” from extreme environments rarely explored by classical AMP discovery [104,105]. Compared with host-associated microbiomes, these environmental datasets potentially provide greater sequence and ecological diversity, whereas host-associated reservoirs may offer candidates that have already evolved within physiologically relevant microbial communities. These approaches should therefore be viewed as complementary rather than competing sources of AMP diversity.

AI also allows researchers to explore biological spaces that would be impractical using classical screening. Wan et al. used deep learning to mine extinct proteomes, screening 10,311,899 peptides for antibiotic activity [38]. This “molecular de-extinction” approach expands AMP discovery beyond living organisms and suggests that evolutionary sequence space may contain antimicrobial motifs that are no longer present in contemporary proteomes.

Other unconventional reservoirs include venom-derived peptides, cryptic peptides encrypted within larger proteins, and host-associated peptide fragments. Guan et al. used venomics AI to explore venom-derived peptide space, showing that venom sequences represent a rich source for antimicrobial discovery [106]. Such approaches indicate that AMPs may be hidden not only in canonical antimicrobial peptide databases but also within larger proteins, ecological interaction networks, and evolutionary archives.

### 5.2. Foundation Models and Peptide Language Models

Protein and peptide language models are increasingly used to represent peptide sequences in high-dimensional embedding spaces. These models can learn patterns from large sequence datasets and support prediction, generation, and optimization tasks. Li et al. developed deepAMP, a peptide-language-based deep generative framework for identifying potent broad-spectrum AMPs [107]. Foundation-model approaches are especially promising because they can capture sequence relationships not obvious from classical descriptors such as charge, hydrophobicity, and amino acid composition. This represents an important shift from descriptor-based prediction toward models capable of learning more complex sequence-context relationships, although improved representation does not by itself guarantee experimentally superior peptides.

A particularly notable example is AMP-Designer, an LLM-based foundation model (built on an AMP-specific GPT architecture, “AMP-GPT”) pre-trained on more than 630,000 peptide sequences from UniProt and fine-tuned through contrastive prompt tuning. Within 11 days, this approach designed 18 de novo AMPs with broad-spectrum activity against Gram-negative bacteria; in vitro validation showed a 94.4% success rate, and two lead candidates combined strong antibacterial efficacy with minimal hemotoxicity, plasma stability, and low resistance-induction potential in murine lung-infection models, with the entire design-to-validation pipeline completed in 48 days [100]. This timeline illustrates how foundation-model-driven design can compress discovery cycles that traditionally took years into a few weeks. A complementary LLM-based framework, ProteoGPT, was pre-trained on 609,216 curated Swiss-Prot sequences and further developed into a pipeline of specialized sub-models—AMPGenix for sequence generation, AMPSorter for AMP classification, and BioToxiPept for cytotoxicity detection [108]. Through this sequential pipeline, more than 82 million non-cytotoxic encrypted AMP candidates were identified by mining, and 4736 de novo candidates were generated; of 196 peptides synthesized and experimentally tested, 143 showed inhibitory activity against *E. coli* or *S. aureus*, with lead candidates demonstrating efficacy in murine thigh-infection models against carbapenem-resistant A. baumannii and MRSA without detectable organ toxicity.

Other LLM-based strategies have been used to identify “encrypted” antimicrobial peptide candidates hidden within larger protein sequences, including a 2025 study that combined a generative protein-sequence LLM with sequence alignment to rapidly discover encrypted peptides active against carbapenem-resistant *A. baumannii* and MRSA, one of which (PL-15) showed efficacy comparable to polymyxin B together with antibiofilm activity.

### 5.3. Generative Design

Generative models now allow AMP research to move from prediction to design. Instead of only classifying existing sequences, generative models can propose new peptides with desired antimicrobial, structural, or physicochemical properties. Wang et al. reported a latent diffusion model that generated diverse AMPs against drug-resistant bacteria, producing broad-spectrum peptides with low cytotoxicity in experimental validation [109]. In this study, AMP-29 showed selective antifungal activity against *Candida glabrata* with in vivo efficacy in a murine skin-infection model, while AMP-24 was active against Gram-negative bacteria and effective in both skin and lung A. baumannii infection models, illustrating that diffusion-generated peptides can progress to in vivo validation [85]. Jin et al. introduced AMPGen, an evolutionary-information-reserved and diffusion-driven model for target-specific AMP design [101]. Of 38 AMPGen-predicted candidates synthesized, more than 80% demonstrated high antibacterial capacity together with sequence diversity and broad-spectrum activity [86]. Gao et al. developed DLFea4AMPGen, which extracts bioactivity-associated features learned by deep learning models and uses them for de novo AMP design; 12 of 16 synthesized peptides showed at least two bioactivities in validation experiments [110].

More recent generative frameworks have focused specifically on pathogen-targeted and property-programmable design. KPPepGen, a knowledge-aware prompt diffusion model published in 2025, incorporates pathogen-specific biological knowledge (via Gene Ontology and pathogen knowledge graphs) to guide diffusion-based generation, allowing simultaneous design of peptides for 56 distinct pathogens; in wet-lab validation against *E. coli* and *S. aureus*, it generated nine novel peptides combining strong antimicrobial activity with low cytotoxicity [102]. A related 2025 framework combined a conditional variational autoencoder with a conditional diffusion model to generate AMPs with programmable physicochemical properties and pathogen-specific targeting, reporting lower predicted MIC values than several other 2025 generative baselines when benchmarked head-to-head [111]. These developments suggest that the field is moving from generic “broad-spectrum” peptide generation toward controllable, pathogen- and property-specific design.

### 5.4. Multi-Objective Optimization

The next step in AI-guided AMP development is not simply to generate more candidate sequences, but to decide how a peptide should be improved. This includes identifying beneficial substitutions, selecting positions for stereochemical modification, preserving antimicrobial activity while reducing toxicity, and optimizing peptides for new activities such as biofilm-relevant endpoints. Recent 2026 studies illustrate this transition from sequence discovery toward modification-aware and function-specific AMP optimization. Zhao et al. developed ADAPT, an AI-based platform trained on D-amino-acid-substituted AMP data to predict which positions in a peptide sequence can be converted to D-amino acids while preserving or improving antimicrobial function [112]. Experimental validation showed that 80% of the AI-designed variants displayed enhanced antibacterial activity, and the lead peptide dR2-1 exhibited improved potency, stability, therapeutic window, membrane-targeting antibacterial activity, and efficacy in a hydrogel-assisted skin infection model [112]. In parallel, ApexGO used a transformer variational autoencoder combined with Bayesian optimization to optimize peptide antibiotic templates through sequence mutation/substitution, synthesizing 100 optimized compounds and achieving an 85% experimental hit rate and a 72% success rate in enhancing activity against Gram-negative pathogens [103].

These two approaches illustrate complementary forms of optimization. ADAPT operates within a chemically defined modification space by identifying advantageous D-amino-acid substitutions, whereas ApexGO explores broader sequence-level changes through mutation and Bayesian optimization. This distinction is important because the former offers greater interpretability and compatibility with established peptide-modification strategies, while the latter provides a wider search space for improving activity. In both cases, however, optimization remains largely driven by antibacterial potency, highlighting the need to incorporate additional properties that determine performance in biofilm-associated infections.

Antibiofilm and antibacterial-oriented AI design is also emerging as an important direction. Pikalyova et al. used an explainable AI/QSAR-guided strategy to design highly potent antimicrobial and antibiofilm peptides within an IDR-1018-derived design space. This study is particularly relevant for biofilm-associated infections because the design objective was not limited to planktonic antimicrobial activity but also included antibiofilm performance. A complementary example is MOFormer (2025), a Pareto-based multi-objective transformer that simultaneously optimizes antimicrobial activity, hemolysis, and toxicity within a single generative pipeline; when extended to a tri-objective scenario incorporating antibiofilm endpoints, MOFormer continued to outperform existing multi-objective baselines, with AlphaFold structure predictions confirming structural reliability of the generated candidates [113]. In a related approach, MPOGAN (2025) introduced a multi-property optimizing generative adversarial network that iteratively learns relationships between peptide sequences and multiple properties including antibacterial potency, hemolytic activity, and biofilm inhibition using a dynamically updated training dataset; experimental validation confirmed that MPOGAN-designed candidates maintained broad-spectrum antibacterial activity while showing significantly reduced hemolysis compared to parent sequences, illustrating how adversarial generative frameworks can balance competing therapeutic objectives in a single optimization cycle [114].

Compared with MIC-centered optimization, these approaches, summarized in Table 2, are more closely aligned with the therapeutic requirements of biofilm-associated infections because they explicitly consider competing endpoints rather than antimicrobial potency alone. However, the three strategies also differ in their level of biological validation: QSAR-guided design focuses on a defined peptide family, MOFormer emphasizes Pareto-based computational optimization across several objectives, whereas MPOGAN combines multi-property generation with experimental validation. These differences make direct ranking difficult but collectively demonstrate a shift toward designing peptides according to therapeutic profiles rather than a single activity metric. From a biofilm perspective, this transition is particularly important because improved planktonic MIC does not necessarily predict inhibition of biofilm formation, penetration into established biofilms, activity against metabolically inactive or persister cells, or eradication of mature biofilm communities. Future AI-guided AMP pipelines should therefore incorporate biofilm-specific endpoints directly during model training, candidate prioritization, and experimental validation rather than treating antibiofilm activity as a secondary property assessed only after peptide generation. Together, these studies show that AI workflows are beginning to optimize AMP candidates across multiple dimensions, including stereochemistry, sequence enhancement, antimicrobial potency, antibiofilm activity, stability, toxicity, and delivery-compatible performance. Overall, the progression from sequence mining to generative design and finally to multi-objective optimization reflects a broader maturation of the field: the principal challenge is no longer simply finding peptides predicted to be antimicrobial but identifying candidates that retain efficacy while simultaneously satisfying the biological and translational constraints required for treatment of complex infections such as biofilms.

### 5.5. Toward Standardized Benchmarking Frameworks for AI-Guided AMP Evaluation

Despite rapid progress in AI-guided AMP discovery, the absence of standardized benchmarking frameworks remains a critical limitation. Most published models are evaluated on self-curated datasets with inconsistent activity thresholds, variable assay conditions, and heterogeneous negative sets, making direct comparison between approaches such as AMP-Designer, ProteoGPT, AMPGen, and KPPepGen practically impossible. A crucial missing element is labeled negative datasets, peptides experimentally confirmed as inactive whose absence limits model robustness and generalizability [115]. Recent initiatives have begun to address this gap. QMAP (2026) introduced a homology-aware benchmark for MIC and hemolytic toxicity (HC50) prediction with predefined test sets, revealing limited progress over six years and poor performance for high-potency peptide regression [116]. ESCAPE (2025) integrated over 80,000 peptides from 27 repositories into a multilabel classification benchmark covering antibacterial, antifungal, antiviral, and antiparasitic activities [117]. PepBenchmark (2026) further addressed data fragmentation by providing a unified framework for peptide machine learning tasks [118]. These efforts reflect a growing consensus that future evaluation pipelines should move beyond binary AMP/non-AMP classification and incorporate experimentally grounded metrics including antimicrobial potency (MIC against standardized ESKAPE panels), hemolytic toxicity (HC50), selectivity index, proteolytic stability, synthesizability, sequence novelty, antibiofilm activity, and in vivo efficacy. Such a multi-metric framework would shift AI evaluation from computational performance toward clinically meaningful benchmarks—a transition the field urgently needs.

For biofilm-oriented AMP development in particular, standardized measurements of biofilm prevention, biomass reduction, viable-cell killing, and activity against established biofilms will be essential if AI-generated candidates are to be meaningfully compared across studies and prioritized for translation.

## 6. Translational Challenges

Although AMP research is expanding rapidly, translation remains difficult. Several AMP or AMP-like candidates have reached clinical testing, but broad translation into approved anti-infective therapies remains limited. Pexiganan, a 22-amino-acid synthetic magainin analogue, illustrates both the potential and difficulty of AMP translation [119]. It was developed as a topical cream for mildly infected diabetic foot ulcers and evaluated in multicenter clinical trials [119]. Earlier studies reported clinical improvement rates broadly comparable to oral ofloxacin, supporting the feasibility of local AMP therapy; however, regulatory approval was not achieved, highlighting the challenge of demonstrating sufficient clinical superiority or added value over existing treatments [119]. LTX-109 is another synthetic antimicrobial peptidomimetic developed for topical use, with clinical studies including nasal *S. aureus* decolonization and non-bullous impetigo. Phase I/IIa and phase II studies showed topical feasibility and antibacterial potential, but broad clinical translation remains limited [120]. Together, these examples show that clinical AMP translation has advanced mainly in topical or local applications, where high local concentrations can be achieved while limiting systemic exposure. This route is favored because many AMPs remain vulnerable to proteolytic degradation, serum binding, salt sensitivity, short half-life, limited tissue distribution, cytotoxicity, formulation constraints, manufacturing cost, and demanding regulatory endpoints.

These same limitations also explain why standard in vitro potency does not always translate into biological or clinical efficacy. Many AMPs show strong activity in standardized broth assays but lose potency in the presence of serum proteins, salts, host proteases, pus, mucus, or biofilm matrices. Mercer et al. emphasized the need for antimicrobial susceptibility testing methods that better predict AMP efficacy under physiologically relevant conditions, such as physiological salt concentrations, serum proteins and wound exudate [34].

This concept is supported by work from De Bleeckere et al., who developed a synthetic synovial fluid model to study biofilm-like aggregates and antimicrobial susceptibility in the context of prosthetic joint infection and showed that joint-like medium can reduce antimicrobial susceptibility compared with conventional laboratory media [121].

A clinically relevant example is PLG0206, an engineered cationic antimicrobial peptide developed for difficult-to-treat and biofilm-associated infections [122]. Rather than being evaluated only in standard planktonic susceptibility assays, PLG0206 was tested in a clinically meaningful ex vivo model using explanted components from chronic total knee arthroplasty prosthetic joint infections. After exposure of the explanted materials to PLG0206 at 1 mg/mL for 15 min, the study reported a mean reduction of approximately 4 log10 in bacterial counts across infected prosthetic components [122]. This type of model is particularly relevant for AMP translation because it evaluates activity against bacteria recovered from real biofilm-associated implant infections, under conditions closer to clinical use than conventional broth MIC assays.

Recent studies further illustrate the importance of evaluating AMPs in clinically relevant wound models, particularly diabetic and infected wound systems. For example, Andrabi et al. evaluated a hybrid-nested microneedle/cryogel platform in biofilm-infected diabetic wound models, including a diabetic swine wound model and type 2 diabetic db/db mice [123]. The microneedle layer delivered vancomycin, the engineered AMP W379, and the pro-angiogenic peptide QK, while the chitosan cryogel layer incorporated magnesium-based metal–organic frameworks to support antioxidant and regenerative functions. This system was designed to mechanically disrupt biofilms and provide localized, sequential therapeutic release. In the diabetic swine biofilm-infected wound model, a single application led to marked biofilm disruption and an approximately 5-log reduction in bacterial burden after 3 days. In type 2 diabetic db/db mice, the microneedle/hydrogel platform promoted wound closure, reaching approximately 80% closure by day 10 and complete closure by day 14, while reducing bacterial burden by approximately 3 logs by day 7 and achieving complete bacterial eradication by day 14 [123].

Da Silva et al. evaluated human β-defensin-2 (hBD-2) delivered through calcium-crosslinked alginate hydrogels in MRSA-infected wounds using streptozotocin-induced diabetic mice [124]. In this model, hBD-2 hydrogels improved wound closure, attenuated inflammation, promoted tissue repair, and reduced MRSA wound burden by 3.68-fold and 8.27-fold on days 3 and 7, respectively, compared with blank hydrogels [124].

Toxicity is another central challenge. Because many AMPs act through membrane interactions, increasing hydrophobicity or amphipathicity may improve bacterial killing but also increase hemolysis or mammalian-cell toxicity. This problem is particularly relevant for lipidated, stapled, or highly hydrophobic peptides. Toxicity evaluation should move beyond hemolysis and standard 2D cytotoxicity assays towards tissue-relevant models. Ex vivo human skin explants are particularly valuable because they preserve tissue architecture, extracellular matrix organization, resident immune components, and barrier properties. Local tolerance can thus be assessed through cell viability, LDH release, histology, cytokine secretion, and tissue repair markers [125]. Such models can help determine whether an AMP remains selective and non-inflammatory in a wound-like tissue environment before progression to in vivo studies.

Resistance development must also be considered. Although AMPs may have a lower propensity for classical single-target resistance, bacteria can evolve reduced susceptibility through surface charge modification, altered membrane lipid composition, capsule production, protease secretion, efflux, stress responses, or biofilm formation [13,14]. Long-term serial passage, resistance frequency assays, and genomic analysis of evolved mutants should therefore be included in preclinical AMP evaluation.

Finally, manufacturing and regulatory issues also remain important. Peptide synthesis can be expensive, particularly for long sequences or chemically modified peptides. Batch reproducibility, purity, stability, formulation, delivery route, and immunogenicity must be addressed before clinical development. For biomaterial-integrated AMPs, regulatory complexity may increase because the product may be considered a combination of drug, device, and material.

## 7. Conclusions

AMPs represent a promising but still under-translated class of antimicrobial agents for biofilm-associated infections. Future progress will depend less on discovering isolated potent sequences and more on engineering complete AMP-based systems that combine antimicrobial potency, antibiofilm activity, selectivity, stability, manufacturability, and clinically relevant delivery. A major priority is to move AMP evaluation beyond standard planktonic MIC assays. Future studies should include biofilm-relevant endpoints, such as prevention of early adhesion, disruption of mature biofilms, killing of tolerant or persister-like cells, regrowth after treatment, and synergy with existing antibiotics. These assays should be performed under physiologically relevant conditions, including serum, salts, wound exudate, proteases, extracellular matrix components, and tissue-compatible models. AI approaches can accelerate this transition by helping to identify not only new peptide sequences, but also the most promising modifications and combinations of properties. Future models should integrate potency, antibiofilm activity, toxicity, stability, solubility, synthesizability, manufacturability, resistance propensity, and compatibility with delivery systems. In this sense, AI should be viewed as a multi-objective optimization tool for designing clinically usable AMPs rather than simply as a sequence-generation approach. Biomaterial delivery should also be considered part of AMP engineering. Polymer choice, peptide loading, immobilization chemistry, release kinetics, surface presentation, and route of administration can strongly influence whether an AMP remains active, selective, and locally available at wound, implant, catheter, or other biofilm-prone interfaces. Overall, the next generation of clinically relevant AMPs will likely emerge from integrated workflows combining rational peptide engineering, AI-guided optimization, biomaterial delivery, physiologically relevant antibiofilm testing, toxicity screening, pharmacokinetic evaluation, and in vivo validation. Successful translation will depend not only on making AMPs more potent, but on making them stable, selective, deliverable, manufacturable, and effective in the complex biological environments in which they are intended to be used.

## Figures and Tables

**Table 1:** Summary of AMP modification strategies and expected functional gains.

Modification Strategy	Main Purpose	Potential Advantages	Main Risks or Limitations	Relevant Sources
D-amino acid substitution	Improve protease resistance	Higher serum stability, prolonged activity	Altered selectivity, reduced activity	Zhong et al. [55], Lu et al. [56], Kalyvas et al. [57]
N-glycine substitution (use of peptoids)	Improve protease resistance	Higher stability, same activity as a regular peptide, easier to synthesize	Complete loss of secondary structure	Wardell et al. [58].
Topological engineering	Conformational stabilization	Increased stability, improved binding, lower degradation	Loss of active conformation	Kalyvas et al. [57], Mitra et al. [60], Pei et al. [61]
Lipidation	Increase membrane affinity	Higher potency, improved retention	Hemolysis and mammalian-cell toxicity	Helny et al. [62], Chen et al. [63], Squittieri et al. [64], Rounds et al. [65]
PEGylation/polymer conjugation	Improve pharmacokinetics	Longer half-life, reduced degradation	Reduced antimicrobial activity	Fontanot et al. [66], Cui et al. [67]
Terminal amidation	Increase charge and stability	Improved helicity and membrane binding	Possible toxicity increase	Wyk et al. [68]
Hydrocarbon stapling	Stabilize α-helices	Higher protease resistance and helicity	Increased hydrophobicity, cost	Meiola et al. [72]
Hybridization	Combine active motifs	Multifunctionality, improved potency	Unpredictable toxicity, folding	Wongchai et al. [75]
Truncation	Identify minimal active core	Lower synthesis cost, reduced toxicity	Loss of potency, stability	Wu et al. [76], Akhash et al. [77].
Metal coordination	Involve bioinorganic chemistry’s tools	Improved activity, better selectivity and modularity	Host toxicity if weak ligation	Song et al. [80], Liu et al. [81].

**Table 2:** Recent AI approaches for AMP discovery and optimization.

AI Approach	Peptide Source or Design Space	Main Contribution	Reference
Transformer VAE + Bayesian optimization	Template peptide optimization	ApexGO for experimentally validated AMP optimization	Torres et al. [103]
Latent diffusion model	Generated AMP sequence space	Diverse AMPs active against drug-resistant bacteria	Wang et al. [109]
Diffusion-driven generative model	Target-specific AMP design	AMPGen for de novo target-specific AMPs	Jin et al. [101]
Feature-learning-based design	Bioactivity-associated peptide motifs	DLFea4AMPGen for multifunctional peptide design	Gao et al. [110]
Venomics AI	Venom-derived peptide space	Discovery of venom-encrypted antimicrobial candidates	Guan et al. [106]
LLM-based foundation model (AMP-GPT)	UniProt-derived peptide space, fine-tuned by contrastive prompt tuning	18 de novo AMPs, 94.4% in vitro hit rate, validated in murine lung infection	Wang et al. (AMP-Designer) [100]
Knowledge-aware prompt diffusion model	56 pathogen-specific design spaces	Pathogen-targeted AMP generation with wet-lab validation	Wang et al. (KPPepGen) [102]
Metagenomics + machine learning	Sludge/environmental metagenomes	>16 million candidate AMPs predicted from waste microbiomes	Xu et al. [104]
Machine-learning mining	Global metagenomes and prokaryotic genomes	AMPSphere catalog of 863,498 non-redundant AMP candidates	Santos-Júnior et al. [37]
Deep-learning prediction	Extinct organism proteomes	Molecular de-extinction of antibiotic peptides	Wan et al. [38]
Peptide language model/deep generative framework	Broad AMP sequence space	deepAMP for broad-spectrum AMP identification	Li et al. [107]

## Data Availability

No new experimental data were generated in this study. Bibliographic data used to generate Figure 3 were retrieved from Web of Science in August 2026 using the search strategy described in the figure caption. All other information discussed in this review is available in the cited literature.
